# Hepatitis E Virus in the Food of Animal Origin: A Review

**DOI:** 10.1089/fpd.2020.2896

**Published:** 2021-06-03

**Authors:** Gianluigi Ferri, Alberto Vergara

**Affiliations:** ^1^ Faculty of Veterinary Medicine, Department of Food Inspection, University of Teramo, Teramo, Italy.; ^2^ Post-Graduate Specialization School in Food Inspection “G. Tiecco,” Faculty of Veterinary Medicine, Department of Food Inspection, University of Teramo, Teramo, Italy.

**Keywords:** hepatitis E virus, RNA, foodborne disease, viral detection, one health, public health

## Abstract

Hepatitis E virus (HEV) is a cosmopolitan foodborne pathogen. The viral agent infects humans through the consumption of contaminated food (uncooked or undercooked). Most cases of infection are asymptomatic and for this reason, this pathology is considered underdiagnosed. Domestic and wild animals are considered natural reservoirs: that is, domestic pig, wild boar, sheep, goat, deer, rabbit, and so on. Therefore, various work categories are at risk: that is, veterinarians, farmers, hunters, slaughterhouse workers, and so on. In these last decades, researchers found a high percentage of positivity to the molecular viral detection in several food matrices included: ready-to-eat products, processed meat products, milk, and shellfish. This review aims to provide an international scenario regarding HEV ribonucleic acid (RNA) detection in several foodstuffs. From this investigative perspective, the study aims to highlight various gaps of the current knowledge about technologies treatments' impact on viral loads. The purpose was also to provide an innovative point of view “One Health”-based, pointing out the strategic role of environmental safety.

## Introduction

Foodborne illnesses caused by viruses are a significant global health problem. In the recent decades, knowledge regarding foodborne viral infections has increased. Furthermore, these pathogens reduce economic growth in many countries in the world (Shirazi *et al.*, 2018).

Data regarding foodborne viral infections change every year. Foodborne viral agents are responsible for 12% of related disease outbreaks, as reported by the European Food Safety Authority (EFSA BIOHAZ Panel *et al.*, 2017).

In the United States, norovirus (NoV) causes ∼58% of foodborne illnesses. However, contaminated food ingestion can also vehicle another important pathogen: hepatitis E virus (HEV).

Indeed, in the last decade there were several outbreaks in the world (Hirneisen *et al.*, 2012).

HEV is transmitted through orofecal route. In general, contaminated water or food of animal origin ingestion (raw or undercooked products) is the main source of infection (Colson *et al.*, 2010).

Furthermore, travels to endemic countries (HEV) pose tourists' health at risk (Guerra *et al.*, 2017; Jemeršić *et al.*, 2019).

HEV belongs to the *Hepeviridae* family and it is classified as *Orthohepevirus A* (Smith *et al.*, 2014). It is a nonenveloped single-strand ribonucleic acid (RNA) virus (Purdy *et al.*, 2017). For this structural characteristic, Johne *et al.* (2016) discovered that virions maintain infectivity for up to 21 days at 37°C and 28 days at room temperature.

Three overlapping open reading frames (ORF) characterize the viral genome. ORF1, ORF2, and ORF3 encode for nonstructural, capsid, and small multifunctional proteins, respectively (Emerson and Purcell, 2003).

*Orthohepevirus* comprises seven genotypes (HEV1–HEV7). HEV1–HEV4 isolated in human hosts, HEV3 in pigs and wild boars (Prpić *et al.*, 2015). HEV5 and HEV6 amplified from wild boars only (Doceul *et al.*, 2016). Recently, an eighth genotype (HEV8) has been proposed as camel variants (Sridhar *et al.*, 2017).

HEV1 and HEV2 are human specific (especially in Asiatic and African continents). These two genotypes infect humans through contaminated water ingestion. This condition is related to an inappropriate wastewaters' management (EFSA BIOHAZ Panel *et al.*, 2017).

HEV3 is widespread in the world (including industrialized countries: European and American continents). It has been derived from both humans and other animal species (domestic swine and wild boar). In general, HEV3 causes sporadic cases in human patients (Kamar *et al.*, 2014; Sayed *et al.*, 2015).

However, EFSA BIOHAZ Panel *et al.* (2017) reported an increasing number of cases of hepatitis E infection (HEV3) among Western European countries. For this reason, many authors suggested that the epidemiological scenario is dynamic.

Consequently, a significant proportion of people in industrialized countries are seropositive (high titers of anti-HEV antibodies Abs), but without typical symptoms of acute hepatitis (Sayed *et al.*, 2015).

HEV4 shows a zoonotic potential. It is considered an Asiatic genotype (Lu *et al.*, 2006; Wang *et al.*, 2021), but Colson *et al.* (2012) isolated this genotype in the European continent too.

In 2013, Yugo and Meng (2013) reported an expanding host range (new animal species found positive for HEV RNA) and demonstrated cross-species infections (Table 1).

**Table 1.** Animal Species Reservoir or Receptive to the Virus (Hepatitis E Virus)

Species	References
Pig	Sasaki *et al.* (2018)
Wild boar	Fredriksson-Ahomaa (2019)
Cattle	Tritz *et al.* (2018)
Sheep	Wang and Ma (2010)
Goat	Tritz *et al.* (2018)
Deer	Prpić *et al.* (2015)
Chicken	Marek *et al.* (2010)
Rat	Lack *et al.* (2012)
Rabbit	Liu *et al.* (2017)
Mongoose	Nakamura *et al.* (2006)
Bat	Drexler *et al.* (2012)
Ferret	Raj *et al.* (2012)
Fish	Batts *et al.* (2011)
Moose	Meng *et al.* (2016)
Camels	Woo *et al.* (2016)
Marine mammals	Montalvo Villalba *et al.* (2017)

There are multiple contact points between humans, wild and domestic animals, and HEV RNA (Nan *et al.*, 2017). This “viral cycle” begins from multiple primary productions: breeding farm/mariculture (especially in extensive realities), slaughterhouses (meat products), processing meat/seafood industries, and wastewaters' management. It concludes with consumers' and environment's health.

Therefore, HEV is a global public health issue that involves humans, animals, and environment. The aim of this article was to purpose a holistic and critical point of view, focusing on the strategic role of dietary transmission that permits HEV virions diffusion. It also explains the impact of food processing technologies on the viral loads and highlights numerous gaps of knowledge concerning HEV contamination in several alimentary industries.

## Categories at Risk

### Veterinary food inspectors, slaughterhouse workers, farmers, and hunters

Veterinary food inspectors, swine farmers, hunters, pork retailers, meat processors, and so on, are routinely exposed to the risk of HEV infection (Montagnaro *et al.*, 2015).

Unsafe raw food, poor storage infrastructure, poor personal hygiene, improper handling methods, and cross-contamination of raw food are just some of the common causes of foodborne diseases that need to be prevented by new measures (Shirazi *et al.*, 2018).

Therefore, they are more likely to be infected with HEV than the general population by 50% (Huang *et al.*, 2018).

Molecular biology (real-time PCR [RT-PCR] assay) and seroprevalence screening assays (immunoglobulin G [IgG] and/or immunoglobulin M [IgM] detection) are gold-standard methods that are used by researchers to evaluate the epidemiological scenario (De Schryver *et al.*, 2015).

Direct contact between infected wild animals (i.e., wild boar) and humans, especially for the above-mentioned work categories, causes high prevalence of anti-HEV Abs.

Carpentier *et al.* (2012) investigated the risk of viral seroprevalence by using enzyme-linked immunosorbent assay. They evaluated seroprevalence in 593 forestry workers (including woodcutters, game or fishing keepers, rangers, and controls) and 421 wild boars. Results evidenced anti-HEV Abs in 31% of the forestry workers and 14% of the wild boars. This study is one of the first papers in which scientists demonstrated that these worker categories are more exposed to HEV virions.

Slaughterhouses have a key role in HEV RNA diffusion. In most of cases, infected swine is asymptomatic, or it presents a self-limiting hepatitis. Therefore, veterinarians cannot identify or suspect positive animals from *ante mortem* evaluation. False-negative animals can enter the slaughterhouse's chain as “healthy.” Consequently, contaminated meat and liver handling diffuses HEV in the entire slaughter lines (Widén, 2016).

In 2019, Milojević *et al.* (2019) screened viral prevalence and respective genotypes in slaughtered pigs in different Serbian slaughterhouses. From the same slaughter line, they collected a total of 345 samples from liver and environmental swabs.

Environmental swabs were collected from different sites/surfaces on the slaughter line having contact with offal and fresh meat (manipulation hooks hanging liver, inspection tables, knives, sharpener, and offal collection containers). The 53% of environmental and 34% of liver samples were positive for the presence of HEV RNA.

Therefore, cross-contaminations start from slaughterhouses and arrive as final products with repercussions on consumers' health.

Krog *et al.* (2019) purpose a new approach of preventive medicine. It is characterized by fecal swabs collected from pigs before their arrival to the slaughterhouses. The detection of HEV RNA (through RT-PCR assay) in feces could be related to HEV positivity in organs. This approach will be able to produce satisfactory results preventing and reducing HEV diffusion in these structures.

In addition, other animal species pose at-risk slaughterhouse workers' health, that is, goats, rabbits (Wang *et al.*, 2016), and sheep (Mesquita *et al.*, 2020).

In rabbit slaughterhouses, Geng *et al.* (2019a) registered high titers of anti-HEV IgG (43.6% male and 47.2% female workers). Seroprevalence was 64.1% among persons with working years >2 and 14.3% among those with working years <0.5 (Geng *et al.*, 2019a). Furthermore, goat farmers, shepherd, and sheep milk cheesemaker workers presented analogous results (Li *et al.*, 2017; Mesquita *et al.*, 2020).

This condition is related to the direct contact with live animals, animal blood (viremia phase), liver, and meat (Wang *et al.*, 2016; Mesquita *et al.*, 2020). Therefore, more attention is required especially during evisceration phase and avoiding bile waste dispersion and cross-contamination regarding muscle masses (Milojević *et al.*, 2019). This evidence highlights one of the crucial HEV pathogenesis principle: the entero-hepato-biliary viral cycle.

This pathogenetic aspect is strictly related to the orofecal viral transmission. It is important to remember that HEV infection is characteristically related to contaminated food ingestion.

Control groups formed by administrative staff, chemists, teachers, and so on, who have no direct contact with biohazard materials, were checked for anti-HEV Abs.

These groups presented high titers ranging between 15% IgG and 18% IgM (Carpentier *et al.*, 2012; Chaussade *et al.*, 2013; Geng *et al.*, 2019a).

Consequently, people living in industrialized countries are generally exposed to the virus owing to the dietary factors (ingestion of contaminated foodstuffs: pork liver, and meat products) (Rivero-Juarez *et al.*, 2017; Delage *et al.*, 2019).

Veterinarians have an important educative role in the implementation of knowledge about zoonoses and their impact on the public health (Bahnson *et al.*, 2001; Poizat *et al.*, 2017). An efficient food safety service has to provide a risk-based surveillance and formative plans (improvement of slaughterhouse's personnel education), as reported by the European Law (EC No. 625/2017) (Huang *et al.*, 2018).

In the near future, strict compliance between private producers and public food safety agencies will improve foodstuffs quality and safety.

### Immunocompromised patients

In immunocompetent patients, HEV is usually asymptomatic (it depends on the genotype) (Weigand *et al.*, 2018). Only a certain percentage of cases present icterus with nausea, fever, abdominal pain, vomiting, and hepatomegaly after an incubation period of 2 to 8 weeks (Park *et al.*, 2016).

Immunocompromised individuals such as organ transplant recipients (Kamar *et al.*, 2008), patients with chronic liver disease (EFSA BIOHAZ Panel *et al.*, 2011), patients affected by HIV infection (Dalton *et al.*, 2009), and those affected by lymphoblastic leukemia (Motte *et al.*, 2012) are some of the categories showing high risk of HEV infection.

These patients usually require the support of blood or blood product transfusions. These administrations can be responsible for interhuman HEV3 and HEV4 transmission (Matsubayashi *et al.*, 2008), because HEV RNA resists blood cell inactivation (Hauser *et al.*, 2014). Therefore, some industrialized countries (Ireland, United Kingdom, Japan, the Netherlands, and Germany) introduced nationwide HEV RNA screenings of blood donations (Domanović *et al.*, 2017).

Most of screened blood samples contained low viral loads and low antibody prevalence (United States 7.7%) (Ditah *et al.*, 2014). Data from United States and Canadian are lower than European countries, that is, IgG 8.7% and IgM 0.4%. Seroprevalence variations depend on the dietary factors (i.e., ingestion of contaminated foodstuffs) (Delage *et al.*, 2019).

High seroprevalence values are also influenced by alimentary and geographical traditions, that is, hunting activities and game meat product consumes. It is largely performed in small communities (anti-HEV immunoglobulin over 30% in Sardinia and Abruzzo regions) (Spada *et al.*, 2019).

Therefore, industrialized countries adopt a new and no alarmistic approach focused on “risk-based decision making.” It purposes molecular screenings of blood, blood products, and foodstuff destined to such vulnerable categories of patients. This system can be used if there is a condition characterized by the low seroprevalence in blood donors (Delage *et al.*, 2019).

## Meat Products

The first evidence of HEV transmission in humans was described in Japan from wild animal meat. These cases were associated with the consumption of uncooked or undercooked pork meat and venison (Matsuda *et al.*, 2003).

In general, contamination comes from the primary production of fresh products, food handling, or water used for food production and preparation (EFSA BIOHAZ Panel *et al.*, 2011).

In contrast to many other foodborne viruses, viral contamination of meat products is not only restricted to the food surface. HEV can also localize in the internal parts of food products (Bouwknegt *et al.*, 2009). Viremia is responsible for HEV virion diffusion in several muscles. Diaphragm muscle may also contain small amounts of pig liver tissue owing to its contiguity. Therefore, sanitary authorities advised to stop using diaphragm muscle in unheated pork products (Bouwknegt *et al.*, 2017).

There is little information concerning HEV survival in food matrices such as ready-to-eat and raw meat products containing swine meat or liver. Consequently, it is necessary to obtain more data about the viral infectious dose from contaminated food consumption.

In general, thermal treatments eliminate the risk of HEV infection. Conversely, there are few studies about the impact of food-processing technologies on viral loads (Sarno *et al.*, 2017).

In North Europe, pork liver sausages are typically uncooked or cooked shortly during processing. Therefore, they still contain virions (Di Bartolo *et al.*, 2012) and vehicle HEV to the final consumers (Kubacki *et al.*, 2017; Giannini *et al.*, 2018) (Table 2).

**Table 2.** Hepatitis E Virus RNA Detection in Meat Products

Country	Meat products	Percentage of HEV RNA detection	References
The Netherlands	255 raw pork sausages		Boxman *et al.* (2020)
	Cervelaat	10.8%	
	Salami	18.5%	
	Metworst	26.1%	
	Snijworst	16.3%	
Switzerland	90 ready-to-eat products		Moor *et al.* (2018)
	Local liver sausages	18.9%	
	Raw meat sausages	5.7%	
Germany	120 meat products		Szabo *et al.* (2015)
	Raw pig liver sausages	22%	
	Raw pig salami	20%	
	Raw wild boar sausages	10%	
Italy	99 traditional pork meat products	0.0%	Montone *et al.* (2019)
	63 wild boar homemade meat and liver sausages	6.3%	
France	394 samples		Pavio *et al.* (2014)
	Typical pig liver sausages	30%	
	Dry salted liver	3%	
	Liver quenelles	25%	
China	107 retail samples		Hao *et al.* (2018)
	Retail pork meats	33%	
	Retail pig livers	8.3%	
	Pig intestines	18.7%	
	Pig spleens	33.3%	
	Pig ureters	26.3%	
China	413 retail samples		Geng *et al.* (2019b)
	Pig livers	6.1%	
	Pig kidneys	3.1%	
	Typical “blood curd”	1.2%	
The Netherlands	537 samples:		Boxman *et al.* (2019)
	Liver	12.7%	
	Liver sausages (“Liverwurst”)	70.7%	
	Liver pate	68.9%	
	Pork chops	0.0%	

HEV, hepatitis E virus; RNA, ribonucleic acid.

Furthermore, traditional and homemade food processing is another crucial aspect. These are typical in Mediterranean European countries (Italy, Spain, France, and Greece) (EFSA BIOHAZ Panel *et al.*, 2011).

In Italy, Istituto Zooprofilattico Sperimentale del Mezzogiorno and Istituto Superiore di Sanità evaluated the detection of HEV RNA in typical and regional homemade meat products. Results are given in Table 2. From these data evaluation, the Italian Public Health Minister purposed new guidelines for a safe domestic production (Montone *et al.*, 2019).

Spain is one of the principal European producers of pork from extensive farming (black Iberian pig) and bushmeat (deer and wild boar). Every year wild boar meat consumption level is ∼3.5 kg per person and it represents an important risk factor for HEV infection, especially in Southern Spain (Pineda *et al.*, 2014).

A representative case was reported by Spanish authors at the Infectious Disease Unit of the Hospital in Cordoba (Rivero-Juarez *et al.*, 2017). A 32-year-old male patient (HIV infected) presented gastroenteric symptoms: malaise, diarrhea, jaundice, vomiting, and fever.

Molecular screenings confirmed HEV RNA detection. The patient's family represented an emblematic example because it comprised wild boar hunters and consumers of game meat (roasted or grilled wild boar meat).

Laboratories also screened two portions of wild boar meat (chap and loin). Both slices were positive with a viral load of 230–500 copies/mL and 155–300 copies/mL for chap and loin, respectively (Rivero-Juarez *et al.*, 2017).

In this case, cooking processes did not completely eliminate HEV, in contrast to the previous studies (Kamar *et al.*, 2014). However, a complete inactivation may also be dependent on the initial viral load and the exact composition of the food matrix (Cook and Van der Poel, 2015).

In the intercontinental scenario, China is the largest pork-producing and consuming country (Geng *et al.*, 2019b).

It is common to consume hot pork (without being frozen). It means that pork products are delivered directly from the slaughterhouse to the retail markets. Pork viscera and blood (i.e., “blood curd” is a popular shortly cooked product) are usually consumed in Chinese popular tradition. These matrices pose at-risk consumers' health (Table 2).

There is a fragmentary knowledge about the resistance of HEV under food processing technologies, that is, fermentation, food aging, curing, drying ( salami, sausages, etc.), smoking, and cooking processes.

Therefore, food product's label should be integrated with information about the potential risk of HEV infection. In this way, the public authorities have to prevent and control possible foodborne outbreaks (Geng *et al.*, 2019b), and protect vulnerable consumers (i.e., patients with preexisting chronic liver disease and transplant recipients) (Delage *et al.*, 2019).

A fundamental solution comes from innovative food technologies application. Emmoth *et al.* (2017) provided the possibility to apply new systems to inactivate viruses (using murine NoV MNV and feline calicivirus FCV as a model for HEV) in swine food matrices.

In particular, they evaluated the effects of high-pressure processing (HPP), lactic acid (LA), and intense light pulse (ILP).

Researchers applied three treatments (HPP, LA, and ILP) to fresh swine liver, dry-cured ham slices, and cold-smoked pork sausages (purchased from local grocery stores). HPP demonstrated efficacy (in the range 300–600 MPa) both on the food surfaces and on internalized viruses. LA and ILP obtained lower viral reduction. Therefore, physical parameters and properties in combination with biochemical ones will encourage new approaches to the food safety concept.

## Milk

The European authorities suggest that milk is a possible source of HEV infection. Therefore, the consumption of unpasteurized and contaminated milk represents a risk for consumers (EFSA BIOHAZ Panel *et al.*, 2017).

The pasteurization process is an important phase in dairy industries. In general, it permits to prevent and control foodborne infection (Huang *et al.*, 2016).

However, in some cases, it is not able to inactivate all viral loads (HEV), and it is possible that consumers infect through pasteurized milk ingestion. Conversely, milk boiling process (100°C for 3 min) provides a complete sterilization (Huang *et al.*, 2016).

Cow's milk has been largely screened in numerous research projects by scientists. However, HEV has been also detected in other dairy species: goat (Long *et al.*, 2017), sheep, and donkey (Demirci *et al.*, 2019) (Table 3).

**Table 3.** International Scenario about Hepatitis E Virus RNA Detection in Milk Products

Continent	Species	Samples	Percentage of HEV RNA detection	References
Europe (Belgium)	Bovine	504 milk samples (collected from 416 dairy farms)	0.0%	Vercouter *et al.* (2018)
Europe (Germany)	Bovine	400 milk samples	0.0%	Baechlein and Becher (2017)
Europe (Turkey)		231 raw milk		Demirci *et al.* (2019)
	Bovine	48 cow milk	29.16% (positive for HEV RNA)	
	Caprine	65 goat milk	18.46% (positive for HEV RNA)	
	Ovine	65 sheep milk	12.3% (positive for HEV RNA)	
	Donkey	53 donkey milk	24.5% (positive for HEV RNA)	
North Africa (Egypt)	Bovine	480 milk samples (collected from 12 farms)	0.2% (positive both for HEV RNA and anti-HEV IgG)	Sayed *et al.* (2020)
			1.6% (positive for anti-HEV IgG)	
Asia (China)	Bovine	140 milk samples	37.1% (positive for HEV RNA)	Huang *et al.* (2016)
	Bovine	416 milk samples	0.0%	Geng *et al.* (2019c)
	Caprine	54 milk samples	74.07% (positive for anti-HEV IgG)	Long *et al.* (2017)

IgG, immunoglobulin G.

There are two different epidemiological realities: on one side industrialized countries, and on the other, developing nations.

In the European Union Member States, HEV RNA and anti-HEV antibodies were not detected (Baechlein and Becher, 2017; Vercouter *et al.*, 2018) (Table 3).

Conversely, in the African (Sayed *et al.*, 2020) and Asiatic continents (Huang *et al.*, 2016; Long *et al.*, 2017; Geng *et al.*, 2019c) it is possible to find contrasting data between rural and industrialized regions (Table 3).

In the rural regions, there are traditional small mixed farms, where different animal species (i.e., bovine, swine, ovine, caprine, and lagomorph) live in direct contact. This condition represents a particular epidemiological scenario that critically improve cross-species infections (Geng *et al.*, 2019c). It is also strictly related to poor hygiene practices “from the stall to the consumers.” Therefore, this factor contributes to increase seroprevalences of anti-HEV IgG Abs.

The role of mammary glands as source of HEV RNA excretion has been investigated in humans too. High titers of anti-HEV antibodies were registered in pregnant women (5.7%, 25/439 mainly owing to the dietary factors: contaminated water and food ingestion) (Alvarado-Esquivel *et al.*, 2014).

High steroid hormone (estrogens) levels (in pregnant women) promote viral multiplication (Singh *et al.*, 2019). Consequently, it is possible to isolate HEV RNA from human breast milk during acute phase of infection (Rivero-Juarez *et al.*, (2016).

In conclusion, it is possible to consider that there is a multifocal sanitary perspective. It begins from healthy farms (through the improvement of zootechnic management during pre- and postdipping phases) and it continues in dairy industries through the introduction of new technologies (i.e., high hydrostatic pressures and ultrasounds) to provide safe food products.

## Shellfish Products

Food handling, freshwaters used in aquaculture, wastewaters, and water used for vegetables and fruit irrigation can be vectors of enteric viruses (i.e., HEV) (EFSA BIOHAZ Panel *et al.*, 2011; Fusco *et al.*, 2019). These viral pathogens arrive to the lamellibranch mollusks' aquaculture farms. Mollusks accumulate and concentrate water microorganisms (bioaccumulation phenomenon) (Krog *et al.*, 2014; La Rosa *et al.*, 2018). Different studies demonstrate the persistence of HEV within shellfish for weeks (Lees, 2000; Loisy *et al.*, 2005; Benabbes *et al.*, 2012).

Therefore, raw edible mollusks' ingestion (i.e., raw oysters) (O'Hara *et al.*, 2018) is a risk for the final consumers' health (Said *et al.*, 2009; Diez-Valcarce *et al.*, 2012). On the other side, thermal treatments (cooking processes) always reduce viral loads (O'Hara *et al.*, 2018).

Mollusks health is strictly related to a correct wastewaters management (bioindicators) (Fiorito *et al.*, 2019). Consequently, environmental safety has repercussion on consumers (Suffredini *et al.*, 2014).

HEV particles are protagonists of a particular “water's cycle” (Fenaux *et al.*, 2019). It begins from virus elimination through human- and animal-infected feces that arrive to the waste treatment plants. Consequently, treated waters can be used in agriculture for fruits and crops irrigation, or they arrive to the sea in mariculture farms.

This cycle concludes with the final consumers, who are infected through contaminated waters, raw mollusks, raw or undercooked meat products, fruits and vegetables ingestion (Fenaux *et al.*, 2019).

Aquaculture farms are frequently localized near harvested areas, that is, urban areas, slaughterhouses and meat preparation industries (O'Hara *et al.*, 2018; Rivadulla *et al.*, 2019), or livestock areas (Barreira *et al.*, 2010). This localization explains the data reported in Table 4.

**Table 4.** Hepatitis E Virus RNA Detection in Shellfish and Water Samples

Screened species	Percentages of HEV RNA positive samples	References
81 *Mytilus galloprovincialis*	14.81%	Mesquita *et al.* (2016)
384 samples		La Rosa *et al.* (2018)
298 *Mytilus* spp.	2.08%	
41 *Siliqua patula*	0.52%	
33 *Chamelea gallina*	0.0%	
12 *Donax* spp.	0.0%	
39 seawater samples	12.8%	
168 samples:		
70 cultured mussels (*M. galloprovincialis)*	28.5%	Rivadulla *et al.* (2019)
35 wild mussels (*M. galloprovincialis*)	37.1%	
31 clams (*Ruditapes* spp.)	16.1%	
32 cockles (*Cerastoderma edule*)	9.3%	
108 mollusk samples	1%	Pupari *et al.* (2019)
23 water samples	4.3%	
63 Pacific oysters (*Crassostrea gigas*)	9.5%	Suffredini *et al.* (2020)
58 clams (*Meretrix lyrata*)	13.8%	

Therefore, waste treatment plants are a critical control point. New surveillance, control measures, and structural implementations could be useful to prevent viral diffusion and infections.

A parallel development of molecular diagnostic assays will provide epidemiological and ecological information regarding HEV circulation (Di Profio *et al.*, 2019).

## Conclusions

HEV detection has been largely studied in meat production chains by numerous international research teams. Furthermore, it has been also detected in other food matrices that is, milk (cow, goat, sheep, and donkey) and mollusks.

Indeed, this review article reports the necessity of further investigations owing to the fragmentary knowledge about the efficacy of food processing technologies on viral loads.

For the future, the World Health Organization (2016) estimates and confirms that numerous foodborne cases of hepatitis E infection (caused by the following genotypes: HEV1, HEV2, and HEV4) will continue to affect endemic countries (African and Asiatic continents).

HEV3 will still be responsible for sporadic infections in Northern America and Europe. Therefore, public health authorities and private producers should enforce and improve monitoring activities in different food production chains. Regional and traditional realities will be crucial viral reservoirs.

Therefore, adequate sanitary measures are required.

For symptomatic human patients, there are two possible approaches to treat and prevent HEV infections.

On the one side, there is a pharmacological approach through ribavirin administration. The British Transplantation Society supports that this molecule provide satisfactory results in transplant recipients (McPherson *et al.*, 2018).

However, ribavirin is not recommended for pregnant women because of numerous adverse and teratogenic effects. Furthermore, emerging variants of HEV influence the efficacy and prognosis of therapies (Todt *et al.*, 2018).

On the other side, vaccination is a valid alternative.

It can be administrated in any epidemiological realities, where several outbreaks occur (endemic nations). In this way, it guarantees immunization for patients at risk.

Hecolin (Xiamen Innovax Biotech, China) is the first registered and licensed vaccine against HEV. It is a protein-based vaccine, and Chinese scientists certified that it induces a valid immune response (Lee *et al.*, 2015).

It provides 100% protection against HEV1 and also cross-protects against HEV4. Therefore, it is strongly recommended for fragile categories.

However, no data are available about cross-protection against HEV3 (World Health Organization, 2016).

Authors underline that is necessary to discover other viral epitopes that can produce adequate immune responses. Furthermore, it is also important that patients' immunoglobulins will provide an efficient cross-protection against different HEV genotypes.

Therefore, this virus still remains an emerging and re-emerging pathogen. It involves the relationships between HEV and multiple factors, that is, human and animal health, alimentary and environmental safety.

Environmental health represents a “critical control point” in foodborne pathogen transmission. On this concept, the European Commission built the new concept of food safety control measures (EC No. 625/2017).

Authors suggest that public authorities should incentivize new system of collaboration between private industries and public research programs. In this way, it will be possible to fill the above-mentioned gaps to obtain a complete point of view regarding HEV.

This article stimulates and inspires new approaches to elaborate data in an innovative way.
